# Palatability of Goat’s versus Cow’s Milk: Insights from the Analysis of Eating Behavior and Gene Expression in the Appetite-Relevant Brain Circuit in Laboratory Animal Models

**DOI:** 10.3390/nu11040720

**Published:** 2019-03-28

**Authors:** Anica Klockars, Erin L. Wood, Sarah N. Gartner, Laura K. McColl, Allen S. Levine, Elizabeth A. Carpenter, Colin G. Prosser, Pawel K. Olszewski

**Affiliations:** 1 Faculty of Science and Engineering, University of Waikato, Hamilton 3240, New Zealand; anica.klockars@waikato.ac.nz (A.K.); elw14@students.waikato.ac.nz (E.L.W.); snm10@students.waikato.ac.nz (S.N.G.); laura.mccoll@waikato.ac.nz (L.K.M.); 2 Department of Food Science and Nutrition, University of Minnesota, St. Paul, MN 55113, USA; aslevine@umn.edu; 3 Dairy Goat Cooperative (NZ) Ltd., Hamilton 3206, New Zealand; Liz.Carpenter@dgc.co.nz (E.A.C.); Colin.Prosser@dgc.co.nz (C.G.P.)

**Keywords:** milk, hypothalamus, nucleus accumbens, reward, appetite, palatability

## Abstract

Goat’s (GM) and cow’s milk (CM) are dietary alternatives with select health benefits shown in human and animal studies. Surprisingly, no systematic analysis of palatability or preference for GM vs. CM has been performed to date. Here, we present a comprehensive investigation of short-term intake and palatability profiles of GM and CM in laboratory mice and rats. We studied consumption in no-choice and choice scenarios, including meal microstructure, and by using isocaloric milks and milk-enriched solid diets. Feeding results are accompanied by qPCR data of relevant genes in the energy balance-related hypothalamus and brain stem, and in the nucleus accumbens, which regulates eating for palatability. We found that GM and CM are palatable to juvenile, adult, and aged rodents. Given a choice, animals prefer GM- to CM-based diets. Analysis of meal microstructure using licking patterns points to enhanced palatability of and, possibly, greater motivation toward GM over CM. Most profound changes in gene expression after GM vs. CM were associated with the brain systems driving consumption for reward. We conclude that, while both GM and CM are palatable, GM is preferred over CM by laboratory animals, and this preference is driven by central mechanisms controlling eating for pleasure.

## 1. Introduction

Milk is a widely consumed, affordable, and highly nutritive food, which serves as a key source of, among others, protein, calcium, potassium, magnesium, and vitamins (especially A and D) in industrialized countries [1,2,3,4,5,6]. In Western societies, cow’s milk (CM) products represent the largest share of dairy available on the market, and cow’s skim milk varieties have become common. However, recent years have generated interest in milk from other species, such as goat’s milk (GM). The use of GM as an alternative to CM has been driven by the findings in humans and laboratory animals showing potential beneficial nutritive consequences of GM intake and differences in physiological responses to GM or CM consumption, (for review, see [7]). For example, Bellioni-Businco et al. reported that individuals with a CM allergy were able to drink five times more GM than CM before the symptoms of an allergic response appeared [8]. In studies utilizing rodent models, Barrionuevo et al. demonstrated that GM increases utilization of copper, zinc, and selenium [9]. Bioavailability of iron and copper was found to be improved in GM-fed rodents suffering from malabsorptive syndrome and in healthy controls [9,10]. Finally, GM improved bone turnover in iron-deficient rats compared to rats fed CM [11,12]. 

Surprisingly, little is known about GM’s acceptance and preference relative to the main dairy product in today’s food environment in the Western world. There is no systematic analysis of propensity to ingest GM and CM or relative palatability of GM vs. CM in either humans or in laboratory animal models. Consequently, our understanding of acceptance and palatability of GM compared to CM is still mainly based on anecdotal evidence and on market availability, both heavily influenced by local cultural or environmental aspects (such as in Western vs. Asian countries) and habituation-driven intake of a specific milk type [13]. This is a major gap in knowledge as palatability affects, among others, the amount of food eaten in a single meal, the rate of consumption, food anticipation, and satiety. It has a profound effect on activity of brain circuits responsible for processing energy intake (including the hypothalamus (HYP) and brain stem (BS)) and reward (such as the nucleus accumbens; NAcc) [14,15,16]. These parameters can, in turn, impact a plethora of mechanisms outside the central nervous system (CNS), via neural and hormonal interactions linking the brain and peripheral tissues [17,18,19]. 

Here, we present for the first time a comprehensive investigation of short-term intake and palatability profiles of GM and CM in laboratory rodent models (mice and rats) using skim milks. We report consumption data on the acceptance (no-choice) and preference (choice) scenarios of calorie-matched milks and milk-enriched solid diets. Consumption data are accompanied by the analysis of expression of appetite-related genes in the HYP and BS, two brain regions predominantly involved in energy balance control, and in the NAcc, a key site that regulates eating for palatability [15,16]. We examined mRNA levels of genes involved in promoting consumption, such as those encoding neuropeptide Y (NPY), Agouti-related protein (AgRP), ghrelin receptor, orexin, opioid peptides/receptors, and gap junction protein, connexin 36 (Cx36). The analysis also included transcripts related to decreased appetite and termination of consumption, such as oxytocin, melanocortin receptors 3 (MC3R) and 4 (MC4R), and proopiomelanocortin (POMC). Typically, presentation of tastants that differ in palatability and composition, among other traits, evokes some changes in expression within this subset of genes, reflecting a different propensity of an animal to ingest specific diets [16,20]. A number of physiological functions by the brain vary with age, including appetite. Weight is typically gained throughout early and middle age, followed by gradual, age-associated anorexia. In line with that, a drive to consume food (and responsiveness to palatability) is high during the earlier stages of life, whereas in aged animals, anhedonia and decreased responsiveness to rewarding diets and to drugs that promote eating for pleasure ensue (e.g., see [21,22,23]). Therefore, in our feeding experiments, we studied rodents that belong to three distinct age groups: adolescents, adults, and aged animals. It should also be noted that rats and most mammals, other than select groups of humans, poorly digest lactose post-weaning. Though lactase activity in adult rats is residual, rats fed as much as 30% lactose in their daily diet from post-weaning to day 98 had normal body growth or body weight course (their body weight was somewhat lower) [24]. Taking it into consideration, however, in this current study we focused on short-term rather than long-term exposure to milk or milk containing chow.

## 2. Materials and Methods

### 2.1. Animals

Male Sprague-Dawley rats and C57Bl mice (all weaned on day 21) used in these studies were single-housed in a temperature-controlled (22 °C) animal facility with a 12:12-h LD cycle (lights on at 07:00). Standard chow (Diet 86, Sharpes Stock Feed, Wairarapa, New Zealand) and water were available ad libitum unless indicated otherwise. The University of Waikato animal ethics committee had approved the procedures (ethics approval numbers: 1020, 1043, and 1057), and they are compliant with the NIH Guide for the Care and Use of Laboratory Animals (NIH Publ., no. 80–23, rev. 1996). Feeding experiments were performed in separate cohorts of animals unless specified otherwise. The age of animals included in the adolescent (5–6 weeks), adult (3–5 months), and aged (25–27 months) categories was based on previous publications pertaining to the aging process in rodents [25]. It should be noted that despite poor digestibility of lactose post-weaning, we did not observe any signs of gastrointestinal discomfort or sickness, which is in line with previous studies showing that rats fed as much as 30% lactose in their daily diet (thus, more than given here) for several weeks displayed good tolerance of the carbohydrate [24].

### 2.2. Episodic Intake of Individually Presented GM and CM in Sated Adult Mice and Rats

We based the protocol on previous studies assessing episodic intake of palatable tastants [26,27,28]. Individually housed mice and rats were accustomed (in homecages) to receiving one of the four isocaloric (0.6 kcal/g) solutions for 2 h/day on 2 days (10:00–12:00) prior to the experiments using their usual 250 mL sized water bottles (used for all bottle scenarios) to avoid neophobia(mice: *n* = 8–9/group; rats: 8–10/group): GM, CM, an energy-equivalent 15% sucrose solution (a reference palatable solution), or a 15% cornstarch (CS) suspension (a negative control for palatability; as CS is insoluble in water, 0.3% xanthan gum was added to this liquid in this experiment as described previously in [29]). On the experimental day, bottles with the solutions (at room temperature) were placed in the cages and water and chow were removed for the 2-h experimental session. Spillage (g) from each individual bottle was recorded before placement into cage. Intakes were measured after 2 h using a digital scale and expressed in grams per gram of body weight. This feeding experiment was conducted in a separate cohort of animals. Composition of the milks are shown in Table 1.

### 2.3. Energy Deprivation-Induced Intake of Individually Presented GM and CM in Mice and Rats

Mice and rats previously exposed in their homecages to GM, CM, cornstarch, and sucrose were deprived of standard chow overnight (food taken away at 16:00). On the next day (10:00), water bottles were removed and replaced with bottles (at room temperature) containing one of the four treatments (mice: *n* = 8–10/group; rats: 7–8/group). Spillage (g) from each individual bottle was recorded before placement into cage. Intakes were measured using a digital scale after 2 h and expressed in grams per gram of body weight. This feeding experiment was conducted in a separate cohort of animals.

### 2.4. Episodic Intake of Individually Presented GM- and CM-Enriched Chow in Sated Adult Mice and Rats

Rats and mice were given episodic access to the chow enriched with GM or CM according to the protocol described above, where, instead of GM or CM, a GM- or CM-enriched chow (see Table 2 for composition of GM and CM chow) was presented for 2 h (10:00). Standard chow pellets were removed during this 2-h meal, but water was left in the cages. Intake of chow pellets (at room temperature) was measured using a digital scale after 2 h and expressed in grams per gram of body weight. In order to assess baseline intake, a control group of animals had a fresh batch of the standard chow placed in the hopper for 2 h (*n* = 7–8/group for both mice and rats). This feeding experiment was conducted in a separate cohort of animals.

### 2.5. Energy Deprivation-Induced Intake of Individually Presented GM- and CM-Enriched Chow in Sated Adult Mice and Rats

Rats and mice previously exposed to GM- and CM-enriched chow (pre-exposure to both chow types was simultaneous) were deprived of standard chow overnight (food taken away at 16:00). On the next day (10:00), animals received either standard chow, GM- or CM-enriched pellets (mice: *n* = 7–8/group; rats: *n* = 8–9/group) at room temperature. Intakes were measured using a digital scale after 2 h and expressed in grams per gram of body weight. This feeding experiment was conducted in a separate cohort of animals.

### 2.6. Episodic Intake of Individually Presented GM and CM in Sated Adolescent and Aged Rodents

Mice and rats aged 5–6 weeks (*n* = 9–11/group for each species) were used in the study on adolescent animals, whereas 25-month old mice and 26-month old rats (*n* = 8–9/group for each species) were used as the aged cohorts. The feeding experiments utilizing individually presented cornstarch, sucrose, GM, and CM solutions followed the protocol described above for the relevant studies in adult sated rodents that received one of the four solutions for 2 h. This feeding experiment was conducted in a separate cohort of animals.

### 2.7. Episodic Intake of GM and CM Presented Simultaneously in Sated Adolescent, Adult, and Aged Rodents

Mice (*n* = 20) and rats (*n* = 21) aged 5–6 weeks were used in the study on adolescent animals, 16–18-week old mice (*n* = 10) and rats (*n* = 12) were included in the study on adults, whereas 25-month old mice (*n* = 12) and 26-month old rats (*n* = 11) were used as the aged cohorts. Adult and adolescent rats and mice had been previously exposed to GM and CM (pre-exposure to both milk types was simultaneous). The aged animals came from the cohorts described above (pt. 2.6), however, a week-long ‘washout’ period was allowed between the previous experiment and this study. First, the animals were accustomed to simultaneously receiving GM and CM as a two-bottle choice (bottles placed next to each other; random order) for circa 1 h per day on two days in their homecage. Then, on the experimental day, chow and water were removed from cages and GM and CM (at room temperature) were given to the animals for 2 h (11:00–13:00). Spillage (g) from each individual bottle was recorded before placement into cage. Intakes were measured using a digital scale after 2 h and expressed in grams per gram of body weight. This feeding experiment was conducted in a separate cohort of adolescent and adult animals. 

### 2.8. Episodic Intake of GM- and CM-Enriched Chow Presented Simultaneously in Sated Adult and Aged Rodents

Mice and rats aged 18–20 weeks old mice (*n* = 8) and rats (*n* = 8) were included in the study on adults, whereas 25-month old mice (*n* = 9) and 27-month old rats (*n* = 10) were used as the aged cohorts. Adult rats and mice had been previously exposed to GM and CM chow (pre-exposure to both chow types was simultaneous). The aged animals came from the same cohort as in the GM/CM chow experiment described above (Section 2.5), however, a two-week-long ‘washout’ period was allowed between the previous experiment and this study. First, the animals were accustomed to receiving simultaneously CM- and GM-enriched chow in a subdivided hopper in their homecages (placement of GM/CM pellets was random; standard chow was removed) for circa 1 h per day on two days. Then, on the experimental day, after removal of standard chow, CM- and GM-enriched pellets (at room temperature) were given to the animals for 2 h (10:00–12:00). Intakes were measured using a digital scale after 2 h and expressed in grams per gram of body weight. This feeding experiment was conducted in a separate cohort of adult animals.

### 2.9. Lickometer-Assessed Preference for Simultaneously Presented GM and CM in Sated Adult Rats

Six 12-week old male rats were housed individually in cages equipped with bottles attached to lickometers (Lafayette Instruments, Lafayette, IN, USA). The animals were previously given GM and CM to prevent neophobia (the pre-exposure was simultaneous). They were accustomed to receiving a choice between GM and CM on two separate days for 30 min (random order of bottles) in lickometer cages. On the experimental day, standard chow and water were removed from the cages and animals were given simultaneous access to GM and CM (room temperature) for 30 min. The number of licks on each bottle was counted and analyzed (Scurry Activity Monitoring software, Lafayette, IN, USA), both as total number of licks as well as number of licks per 5-minute interval. We also assessed the cluster number (number of bouts of licking—each bout was defined as continuous licking interspaced by no more than 0.5 s between each other) and an average cluster length (bout duration measured in seconds) of GM vs. CM. This feeding experiment was conducted in a separate cohort of animals.

### 2.10. 72-h Cumulative Intake of Simultaneously Presented CM- and GM-Enriched Chow in Adult Rats

First, the animals were accustomed to receiving two types of chow pellets (room temperature) simultaneously in a subdivided hopper in their homecage (placement of pellets was random) for circa 2 h per day on two days. On the experimental day 1 (17:00), animals received a choice of either standard/CM chow (*n* = 9), standard/GM chow (*n* = 10), or GM/CM chow for 72 h (pellets were exchanged daily; *n* = 16). Cumulative 72-h intakes were recorded in grams. This feeding experiment was conducted in a separate cohort of animals.

### 2.11. Effect of 24-h CM vs. GM Consumption on Feeding-Related Gene Expression in the Brain Circuit

In order to assess the effect of 24-h intake of GM and CM solutions on the expression of feeding-related genes in the brain, mice were given CM or GM (at room temperature) as the only tastant (starting at 10:00). Animals given water served as baseline controls. At 10:00 on the subsequent day (thus, 24 h after milk presentation), the animals were sacrificed via cervical dislocation. Brains were dissected out and the hypothalamus, nucleus accumbens, and brain stem excised and stored in RNAlater at −80 ° C until further processing. This experiment was conducted in a separate cohort of animals.

Tissues were homogenized in Trizol (Ambien), 1 mL per 0.1 g tissue. 0.2 mL chloroform was added and samples were centrifuged at room temperature for 10 min at 10,000× *g*. The clear phase containing RNA was isolated and 0.5 mL of isopropanol was added. RNA was precipitated in an ice bath for 10 min then centrifuged at 4 °C for 20 min at 10,000× *g*. Aqueous phase was removed from the pellets, which were then resuspended in 0.3 mL of ethanol and centrifuged at 4 °C for 10 min at 10,000× *g*. Liquid was removed and pellets were air-dried.

Pellets were dissolved in 8 µL DEPC water and 1 µL DNAse buffer (dNature). Samples were then incubated with 1 µL DNAse (dNature) at 37 °C for 30 min. DNAse was inactivated via addition of stop buffer (dNature) and incubation at 67 °C for 10 min. Removal of DNA was confirmed via PCR using HOT FIREPol Blend Master Mix (dNature), followed with agarose gel electrophoresis. Concentrations of RNA were measured with a nanodrop. 

cDNA was synthesized from RNA samples with iScript Advanced cDNA synthesis kit (BioRad). Synthesis of cDNA was confirmed with PCR followed by agarose gel electrophoresis. 

Quantitative RT-PCR (qPCR) was used to determine relative expression levels of housekeeping genes (ActB, GAPDH, β-tubulin) and of genes of interest. Reactions contained 4 µL of 25 ng/μL sample cDNA, 1 µL of each forward and reverse primers (5 µM), 10 µL iTaq Universal SYBR Green Supermix (BioRad) and 4 µL MilliQ water. qPCR experiments were performed in duplicates alongside negative controls of MilliQ water for each primer pair. Amplification protocol was initiated at 95 °C for 15 min, followed by 45 cycles of 15 s at 95 °C, 15 s at the primer-specific annealing temperature and 30 s at 72 °C. Primers used: GADPH F: 5′-AAGGTCATCCCAGAGCTGAA-3′, R: 5′-CTGCTTCACCACCTTCTTGA-3′; ActB F: 5′-AGTGTGACGTTGACATCCGT-3′, R: 5′-TGCTAGGAGCCAGAGCAGTA-3′; POMC F: 5′-GACACTGGCTGCTCTCCAG-3′, R: 5′-AGCAGCCTCCCGAGACA-3′; Agrp F: 5′-GGATCTGTTGCAGGAGGCTCAG-3′, R: 5′-TGAAGAAGCGGCAGTAGCACGT-3′; NPY F: 5′-GGTCTTCAAGCCGAGTTCTG-3′, R: 5′-AACCTCATCACCAGGCAGAG-3′; MC4R F: 5′-CTTATGATGATCCCAACCCG-3′, R: 5′-GTAGCTCCTTGCTTGCATCC-3′; β-tubulin F: 5′-CGGAAGGAGGCGGAGAGC-3′, R: 5′-AGGGTGCCCATGCCAGAGC-3′; GHSR F: 5′-TCCGATCTGCTCATCTTCCT-3′, R: 5′- GGAAGCAGATGGCGAAGTAG-3′; ORX F: 5′-GCCGTCTCTACGAACTGTTGC-3′, R: 5′-CGCTTTCCCAGAGTCAGGATA-3′; OXT F: 5′-CCTACAGCGGATCTCAGACTG-3′, R: 5′-TCAGAGCCAGTAAGCCAAGCA-3′; OXTR F: 5′-TCTTCTTCGTGCAGATGTGG-3′, R: 5′-CCTTCAGGTACCGAGCAGAG-3′; PENK F: 5′-CGACATCAATTTCCTGGCGT-3′, R: 5′-AGATCCTTGCAGGTCTCCCA-3′; DYN F: 5′-GACAGGAGAGGAAGCAGA-3′, R: 5′-AGCAGCACACAAGTCACC-3′; GHRL F: 5′-CCATCTGCAGTTTGCTGCTA-3′, R: 5′-GCTTGTCCTCTGTCCTCTGG-3′; MOR F: 5′-CCTGCCGCTCTTCTCTGG-3′, R: 5′-CGGACTCGGTAGGCTGTAAC-3′; KOR F: 5′-CACCTTGCTGATCCCAAAC-3′, R: 5′-TTCCCAAGTCACCGTCAG-3′; PNOC F: 5′-AGCACCTGAAGAGAATGCCG-3′, R: 5′-CATCTCGCACTTGCACCAAG-3′; ORLP F: 5′-ATGACTAGGCGTGGACCTGC-3′, R: 5′- GATGGGCTCTGTGGACTGACA-3′; GLP1R F: 5′-ATGGCCAGCACCCCAAGCCTCC-3′, R: 5′-TCAGCTGTAGGAACTCTGG-3′; Cx36 F: 5′-CCAGTAAGGAGACAGAACCAGAT-3′, R: 5′-GATGATGTAGAAGCGGGAGATAC-3′.

### 2.12. Data Analysis

Analyses of qPCR data utilized BioRad CFX Manager software (BioRad); one-way ANOVA followed by Bonferroni’s test with the correction for multiple comparisons was used, with *p* < 0.05 set as criterion of statistical significance. Feeding data from studies comparing two groups were analyzed using a *t*-test, whereas comparisons between three or more groups were done with ANOVA followed by Bonferroni’s post-hoc test, with differences considered significant when *p* < 0.05. 

## 3. Results

**Figure 1:**
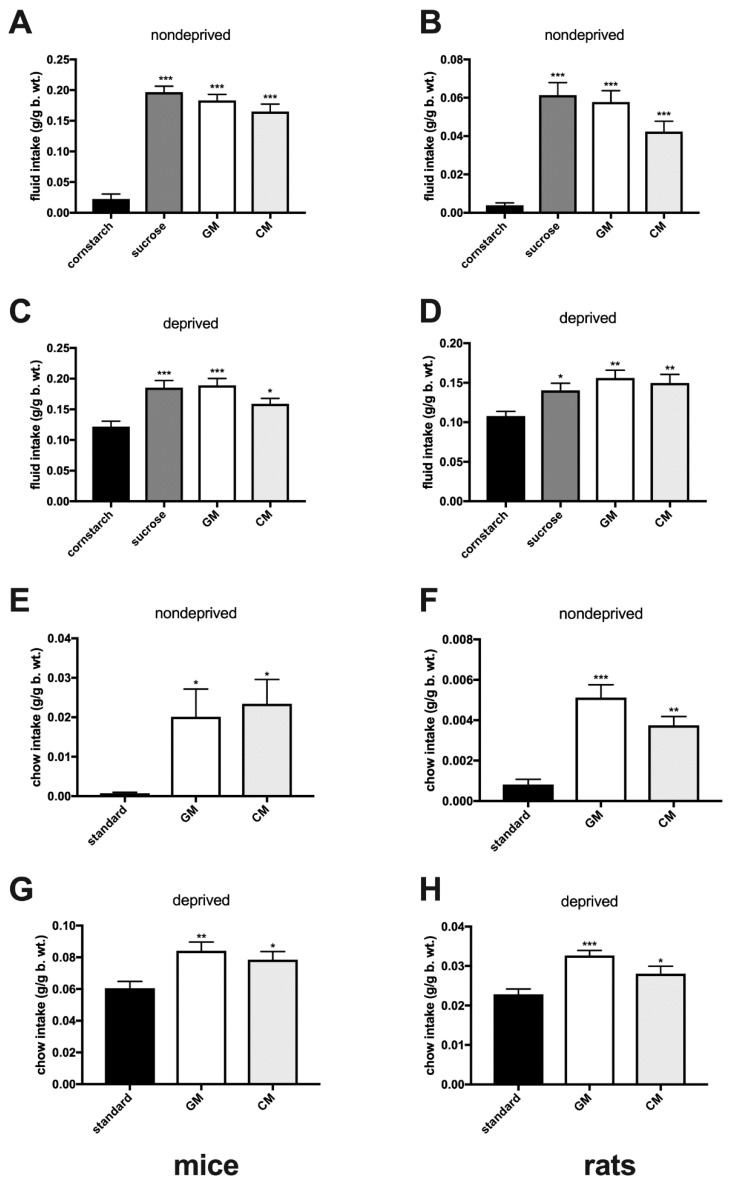
Episodic 2-h consumption of individually presented (acceptance) cornstarch, sucrose, GM, and CM isocaloric solutions (**A**–**D**), and of standard, GM- and CM-enriched chow (**E**–**H**) in sated (nondeprived) and energy-deprived mice (left panel) and rats (right panel).*, *p* ≤ 0.05; **, *p* ≤ 0.01; ***, *p* ≤ 0.001.

**Figure 2:**
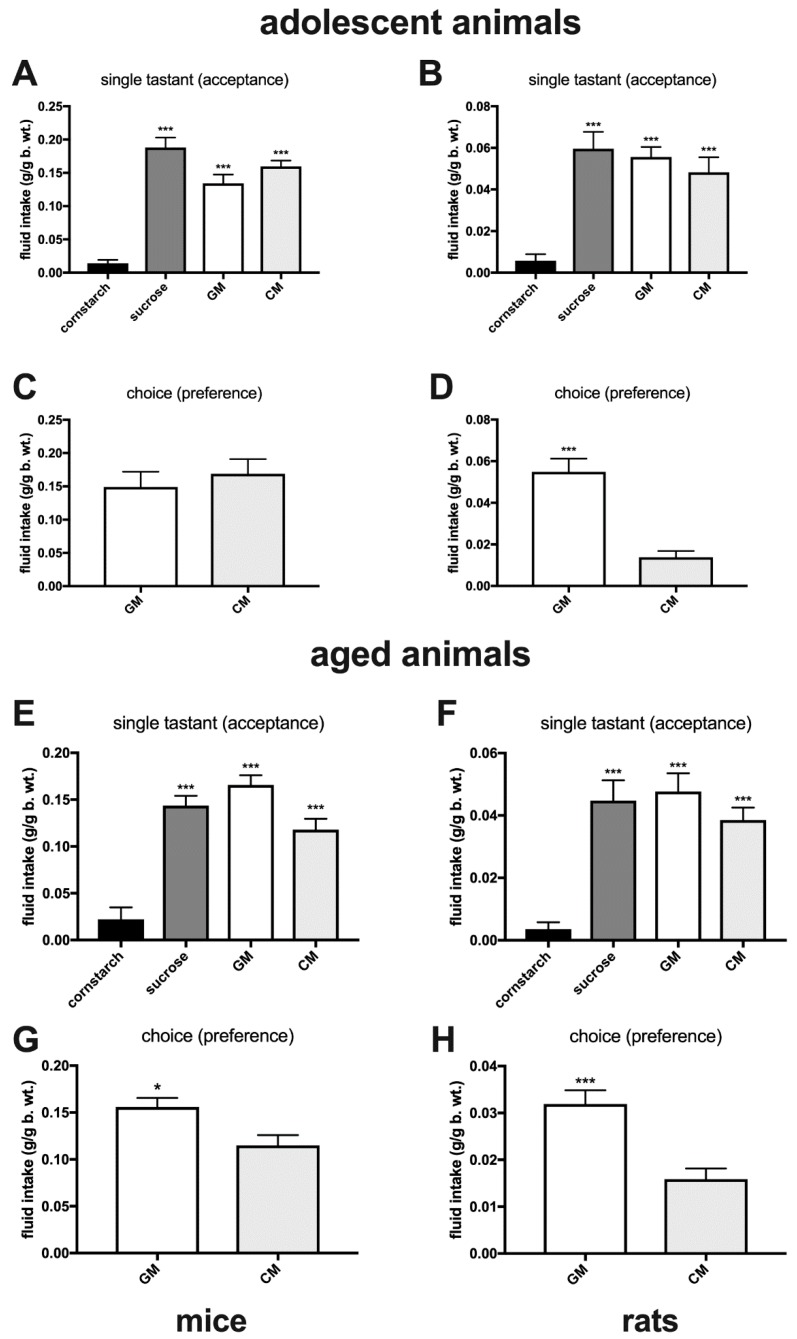
Episodic 2-h consumption of individually presented cornstarch, sucrose, GM, and CM isocaloric solutions (**A**,**B**,**E**,**F**: acceptance) and simultaneously given GM and CM (**C**,**D**,**G**,**H**: preference) in adolescent and aged sated mice (left panel) and rats (right panel). *, *p* ≤ 0.05; ***, *p* ≤ 0.001.

In the non-choice acceptance tests, sated adult mice and rats showed very low levels of consumption of a ‘bland’ cornstarch emulsion, whereas intakes of the GM (mice, *F*(3,30) = 62.8, *p* < 0.001; rats, *F*(3,32) = 25.5, *p* < 0.001) and CM (mice, *p* < 0.001; rats, *p* < 0.001), as well as of the sucrose solution (mice, *p* < 0.001; rats, *p* < 0.001), were several times higher than of cornstarch. Energy-deprived animals had a higher baseline intake of cornstarch, but consumed significantly more sucrose (mice, *F*(3,32) = 9.77, *p* ≤ 0.001; rats, *F*(3,26) = 5.5, *p* = 0.039), GM (mice, *p* < 0.001; rats, *p* = 0.0023), and CM (mice, *p* = 0.034; rats, *p* = 0.0083; Figure 1A–D). Similarly, both deprived and sated adult individuals ate more GM- and CM-enriched pellets than standard chow (sated mice: *F*(2,19) = 5.9, GM, *p* = 0.029 and CM, *p* = 0.011; sated rats: *F*(2,19) = 20.5, GM, *p* < 0.001 and CM, *p* = 0.0011; deprived mice: *F*(2,19) = 6.5, GM, *p* = 0.0058 and CM, *p* = 0.034; deprived rats: *F*(2,22) = 10.8, GM, *p* < 0.001 and CM, *p* = 0.0442; Figure 1E–H). Adolescent and aged sated mice and rats (Figure 2A,B,E,F) given episodic 2-h access to one of the solutions, consumed more GM (adolescent mice, *F*(3,35) = 42.7, *p* < 0.001; rats, *F*(3,36) = 16.9, *p* < 0.001; aged mice, *F*(3,29) = 31.2, *p* < 0.001; rats, *F*(3,29) = 18.9, *p* < 0.001), CM (adolescent mice, *p* < 0.001; rats, *p* < 0.001; aged mice, *p* < 0.001; rats, *p* < 0.001) and sucrose (adolescent mice, *p* < 0.001; rats, *p* < 0.001; aged mice, *p* < 0.001; rats, *p* < 0.001) than cornstarch. 

**Figure 3:**
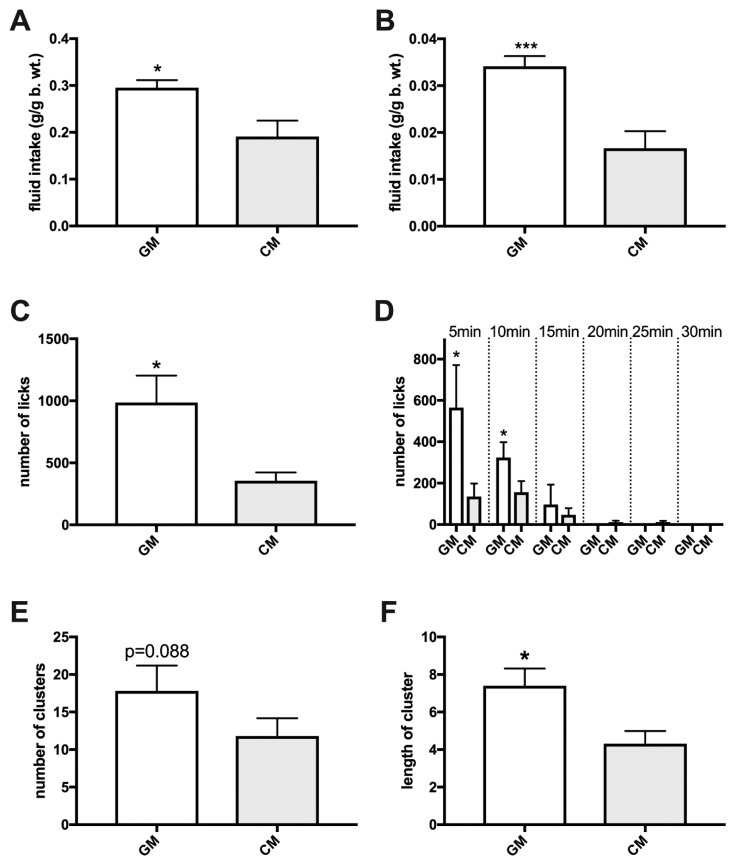
Episodic consumption of simultaneously presented GM and CM over 2-h in sated mice (**A**) and rats (**B**), lickometer activity during a 30-min exposure ((**C**): 0–30 min; (**D**): 5-min intervals), the number of GM over CM licking bouts (cluster number) (**E**), and the cluster length(s) of each GM and CM bout (**F**) in sated rats. *, *p* ≤ 0.05; ***, *p* ≤ 0.001.

**Figure 4:**
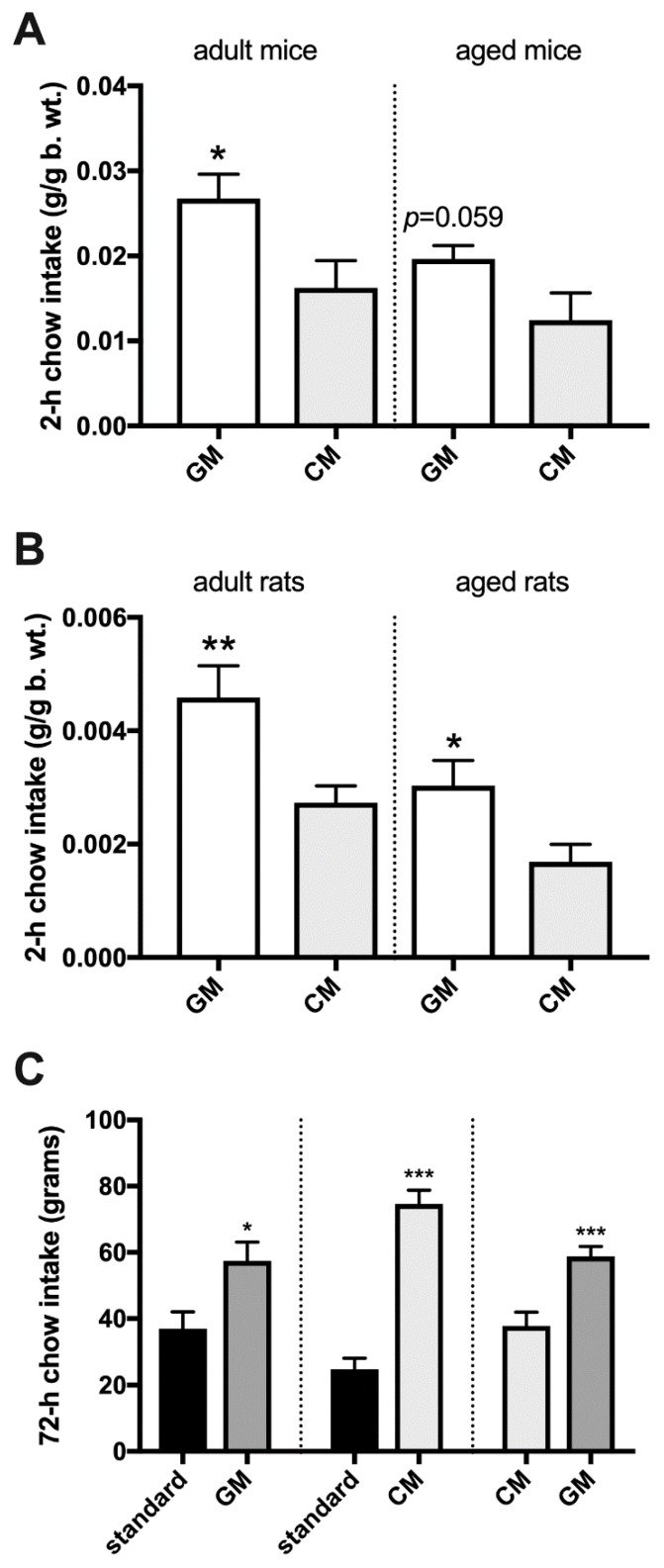
Consumption of simultaneously presented GM- and CM-enriched chow in adult and aged sated mice (**A**) and rats (**B**) over 2 h and simultaneously presented pellets (standard vs. GM; standard vs. CM, and GM vs. CM) over 72 h in adult rats (**C**).*, *p* ≤ 0.05; **, *p* ≤ 0.01; ***, *p* ≤ 0.001.

When given a 2-h episodic choice between GM and CM, all age cohorts of rats (adolescent, *p* < 0.001; adult, *p* < 0.001; aged, *p* < 0.001) and adult and aged mice (*p* = 0.012 and 0.011, respectively) preferred GM (Figure 2C,D,G,H and Figure 3A,B). During a brief, 30-min exposure to both GM and CM in cages equipped with lickometers, adult rats exhibited a more robust response to GM cumulatively over that period (*p* = 0.01) as well as during the first (*p* = 0.037) and second (*p* = 0.05) 5-min time interval of the meal (Figure 3C,D). There was a trend approaching significance (*p* = 0.088) toward an increase in the cluster number (number of licking bouts) of GM over CM, and a significantly greater cluster length of each GM than CM bout (*p* = 0.022; Figure 3E,F). In choice experiments involving GM- and CM-enriched chow, adult and aged rats (*p* = 0.009 and 0.023, respectively) and adult mice (*p* = 0.028) preferred GM chow, whereas in aged mice, a trend toward GM preference was detected (*p* = 0.059) (Figure 4A,B). Adult rats given a 72-h uninterrupted access to a choice between GM and CM chow preferred GM chow (*p* < 0.001), while both GM (*p* = 0.015) and CM pellets (*p* < 0.001) were preferred over standard food during a similar time of exposure (Figure 4C).

**Figure 5:**
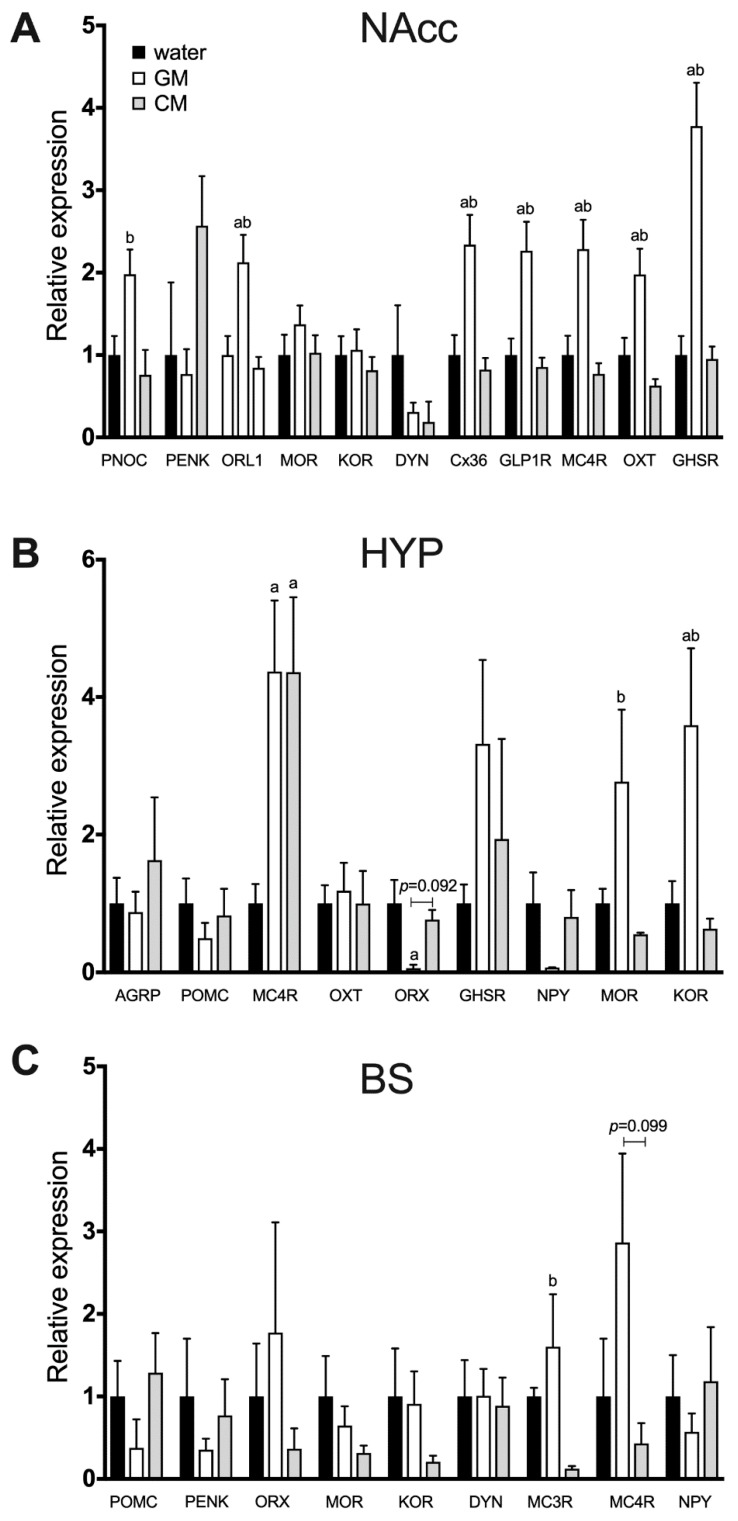
Relative expression of feeding-related genes in the nucleus accumbens (**A**), hypothalamus (**B**), and brain stem (**C**) of mice maintained for 24 h on GM or CM. Water served as a baseline tastant. ^a^—significantly different from the water group; ^b^—significantly different from the CM group. Analysis performed with ANOVA followed by Bonferroni’s test and corrected for multiple comparisons.

Real-time PCR analysis after consumption of the two milk formulations (GM: 19.27 +/− 0.18 g; CM: 18.44 +/− 0.17 g) revealed that GM upregulated in the nucleus accumbens PNOC (*p* = 0.0164), ORL1 (*p* = 0.0042), Cx36 (*p* = 0.0017), GLP1R (*p* = 0.0015), MC4R (*p* = 0.002), OXT (*p* < 0.001), and GHSR (*p* < 0.001) genes, whereas mRNA levels of PENK were lower (though it did not reach significance with a *p* value of 0.01), compared with CM consumption. In the hypothalamus, MOR (*p* = 0.045) and KOR (*p* = 0.017) transcript levels were higher after GM consumption, and in the brain stem there was a trend toward upregulation of the MC4R (*p* = 0.099) and the MC3R was upregulated (*p* = 0.0275; Figure 5). Compared to water controls, in the nucleus accumbens, GM affected expression of ORL1 (*p* = 0.012), Cx36 (*p* = 0.0052), GLP1R (*p* = 0.0042), MC4R (*p* = 0.0053), OXT (*p* = 0.0149), and GHSR (*p* < 0.001); in the hypothalamus, ORX (*p* = 0.0164), KOR (*p* = 0.0399), and MC4R (*p* = 0.0403). On the other hand, hypothalamic expression of the MC4R gene was elevated by CM intake (*p* = 0.041; Figure 5).

## 4. Discussion

Enhanced motivation to eat in the absence of an immediate need to replenish calories or continuation of a meal beyond levels that restore energy balance typically occur when an individual is given access to food that is highly palatable. In laboratory animal models, similarly to what is observed in humans, a variety of tastants are perceived as palatable. Those include ingestants whose palatability is derived mainly from the flavor and/or postabsorptive effects of either a single macronutrient (e.g., sucrose-sweetened solutions) or from the complex contribution of multiple nutritive components (e.g., in meat rich in protein and fat) [30,31,32]. Calorie density of food (especially when coupled with high energy needs of an organism) is an additional factor that affects the liking of and preference for a given food [15,33]. 

The current set of studies show that both GM and CM and milk-enriched solid diets are highly palatable. In no-choice acceptance paradigms, energy non-deprived rats and mice of all age groups (adolescent, adult, and aged) consumed GM and CM as avidly as the calorie-matched 15% sucrose solution (used here as a positive control for a highly palatable tastant in rodents (for review, see [30]), while ingesting only minimal amounts of the ‘bland’ cornstarch. A similar phenomenon was observed in energy-deprived animals, although the amount by which GM, CM, and sucrose intakes exceeded that of cornstarch was not as pronounced as in sated rodents. That was due to the vigorous energy deficit-driven consumption of cornstarch and a ‘ceiling effect’ that prevents ingestion of large amounts of the solutions during the brief refeeding period. Importantly, GM and CM enrichment of laboratory chow stimulated intake in both hungry and sated animals well above the level of standard pellets. It indicates that both GM- and CM-derived palatability is a generalized phenomenon, not limited to liquid milks, but extending to solid foods that contain milk powder. This is in concert with the ability of other palatable tastants (including, but not limited to, fat, sucrose, and select amino acids) to have a positive gustatory effect when presented as a component of both liquid and solid foods [34]. The fact that not only adolescent and adult animals, but also the aged ones, readily consume GM and CM suggests that age-related decline in hedonic processing [22,35,36,37] does not completely abolish a drive to eat milk-based diets. Instead, a slightly depressed intake of GM and CM at an old age parallels that reported for sweet solutions, as shown here and by other authors [38,39,40]. This finding is particularly relevant from the standpoint of being able to use palatable GM or CM as nutritionally superior alternatives to, e.g., sucrose-sweetened tastants in aged individuals [23]. That adolescent rodents also consume large quantities of both milk types indicates that prolonged dietary habituation is not required to develop the liking of either GM or CM. In fact, the amounts of GM and CM ingested by juveniles were as high as the volume of sucrose (readily consumed in large quantities by young animals, e.g., see [41]) even though the individuals had had only two brief exposures to these solutions prior to the experiment. 

The single-tastant scenarios above strongly suggest a high acceptance level for both GM and CM indicating they are palatable, but as these no-choice paradigms produced fairly similar feeding responses, choice studies were needed to define relative preference for these two milk types. Simultaneous 2-h exposure to two bottles containing GM and CM showed that adult and aged mice and rats as well as adolescent rats exhibit a marked preference for GM (adolescent mice were the only cohort in which GM and CM were iso-palatable). The preference for GM did not appear to be related to whether the animals’ pre-exposure to the specific diets was simultaneous (such as in adolescents and adults) or sequential (aged rodents). This finding was further expanded by employing the 30-min lickometer analysis in adult rats. It showed approximately four times as many licks at the bottle containing GM compared to CM during the first 5 min of the meal, and twice as many licks at the GM bottle in the subsequent 5-min interval. Overall, the licking activity at both bottles occurred within the same timeframe with neither milk type being ingested in a prolonged fashion. It increases our confidence in that motivation to consume palatable GM rather than maintenance of a meal (due to, e.g., delayed satiation [42]) is the main reason for avid intake of GM. The analysis of the licking bouts provides additional support for this notion. The cluster number (total number of bouts) neared significance for GM, possibly reflecting the incentive motivational properties of the food stimulus; importantly, the relationship of motivation and this measure reflects post-ingestive negative feedback [43,44,45,46,47]. On the other hand, the average cluster length—significantly greater for the GM formulation—typically parallels the hedonic properties (mainly, orosensory pleasure) of ingestive stimuli (as reviewed, e.g., in [45]). In this case, it is the length of clusters that appears to be the main driver for the preference for GM. A good example of the significance of licking bout length versus number in the context of neural regulation of food intake comes from studies on the endogenous opioid system. Ostlund et al. found that mu opioid receptor (MOR) knockout (KO) mice show alterations in sucrose licking: while energy-deprived wild-type mice increased burst length, relative to the nondeprived condition, this aspect of licking was insensitive to changes in food deprivation in MOR KOs. The rate of sucrose and sucralose licking in KOs was lower than in wildtype animals, providing evidence that the MOR was involved in processing palatability [48]. Mendez and colleagues reported that proenkephalin (PENK) KOs given a sucrose solution exhibited fewer bouts of licking (though the length did not differ) than wild type controls, indicating a diminished motivation to eat [46]. Finally, studies on the involvement of nociceptin/orphanin FQ (NOC) revealed that NOC administration initiates new bouts of licking for sweet solutions, which is in line with the notion of its potential relationship to motivational aspects of feeding. Interestingly, energy-deprived NOP KO mice given sucrose showed longer bouts of licking than wild types, suggesting that, under hungry conditions, NOC may also affect hedonics of consumption [49].

The notion that satiety is not delayed by GM intake is supported by the experimental work exploring satiating effects of a CM- versus GM-based meal in humans. In their study, Rubio-Martín et al. presented healthy adults with GM-based or CM-based breakfast after an overnight fast and obtained blood samples and appetite ratings from the subjects just before and up to 5 h after completion of the meal. They found that that the ‘desire to eat’ rating was significantly lower and hunger rating tended to be lower after the GM breakfast. Interestingly, the area under the curve (AUC) for a satiety hormone GLP-1 was inversely associated with the AUChunger and AUCdesire-to-eat after the GM meal [50]. 

The aforementioned data obtained in human observations combined with the current results of our experiments in animal models suggest that even though composition differences between GM and CM are relatively minor, they are sufficient to significantly affect appetite-related parameters. It remains to be elucidated whether these effects are produced by a specific macronutrient component, a combination of nutritive components, and/or some physico-chemical characteristics of each milk type (e.g., micelle structures in GM vs. CM differ in diameter, hydration, and mineralization) [51]. 

The analysis of mRNA levels of feeding-related genes sheds more light on neural processing underlying enhanced preference for GM over CM. One of the most striking outcomes is the fact that, unlike in the NAcc, which showed an increase in multiple mRNA profiles after GM over CM, there are relatively few significant differences in gene expression in the hypothalamus and brain stem. Those two brain areas serve as the foundation for the control of energy homeostasis and consumption-related changes in the internal milieu associated with plasma osmolality, stomach distension, and defense from exposure to food-borne toxins [52]. In this network, the brain stem acts as the relay station between the periphery and the central nervous system, whereas the hypothalamus plays a endocrine role (by releasing, e.g., anorexigenic hormones, such as oxytocin (OXT) via the neurohypophysis) and innervates a number of central target sites (it includes the reciprocal connectivity with the brain stem, as well as multiple pathways with, among others, nigrostriatal and hippocampal structures). It is noteworthy that, despite the same level of intake of GM and CM over the 24-h period, the hypothalamic expression of NPY and orexin (ORX) was lower in the GM group. Both ORX and NPY in the hypothalamus enhance consumption chiefly by increasing hunger and motivating intake of energy-dense tastants [53,54]. Thus, these data suggest that enhanced preference for GM over CM does not stem from the stimulation of neural mechanisms that lead to hunger-driven feeding. In line with the aforementioned conclusion from feeding experiments that the increased preference for GM vs. CM in choice scenarios is unlikely to be related to suppressed satiety signaling, we found that the brain stem expression of satiation promoting melanocortin receptors [55,56] is elevated after consumption of GM (it remained the same in the hypothalamus). This change in the receptor mRNA level coupled with the lack of a difference in the melanocortin ligand precursor gene expression (proopiomelanocortin, POMC) as well as in the anorexigenic OXT gene [28,57] suggests the lack of impairment in central satiety processing after GM (and, surprisingly, even a somewhat greater sensitivity of the molecular network promoting satiety in response to GM consumption). 

Interestingly, the hypothalamic genes whose expression was elevated by GM intake were those encoding the MOR and kappa (KOR) opioid receptors (MOR and KOR brain stem and accumbal mRNA levels were also higher, though the difference did not reach statistical significance). Furthermore, in the NAcc, we found overexpression of genes coding for opioid-related NOC and this peptide’s receptor, ORL1. Opioid receptors are directly implicated in the regulation of feeding for reward [14,42]. They are part of a dispersed network that includes the NAcc as one of the key sites mediating hedonic aspects of eating behavior. They are also expressed throughout the ‘homeostatic’ components of the feeding-related circuit [16], including the hypothalamus and brain stem, where they are theorized to promote excessive consumption of palatable tastants by delaying meal termination. The magnitude at which opioid receptor agonists, such as butorphanol tartrate, dynorphin and beta-endorphin, stimulate consumption parallels the relative palatability of foods [14,58]. Conversely, opioid receptor antagonists, e.g., naltrexone and naloxone, are particularly effective at reducing intake of tasty ingestants [59]. Hence, higher expression of the MOR and KOR mRNA after GM is in line with the observed preference for the GM over CM. Changes in expression of additional NAcc genes that underscore the functional relationship between GM intake and reward processing include upregulation of Cx36 mRNA, as Cx36 ensures proper synchrony of dopaminergic pathways [60], and of the growth hormone secretagogue receptor (GHSR) mRNA, considering that the GHSR in the NAcc has been found to mediate hedonics of ingestive behavior [61]. Again, as in the case of the hypothalamic gene expression analysis, genes encoding molecules that promote satiety—such as OXT, melanocortin receptor 4, and glucagon-like peptide-1 receptor [62]—were upregulated after GM, which points to the heightened reward processing rather than impaired satiation as the factor propelling preference toward GM over CM.

## 5. Conclusions

We conclude that, in laboratory animal models, GM and CM are highly palatable when presented as liquids and as components of solid diets. Diet choice paradigms reveal preference for GM over CM in mice and rats belonging to different age groups. Feeding studies and analyses of gene expression in the feeding-relevant brain circuit point to feeding reward as the main factor underlying preference for GM. While the current studies draw on the laboratory animal experimental approaches, they have a translational impact relevant to our understanding of the consequences of GM consumption in humans. This outcome is particularly important as, globally, likely more people drink GM than CM. GM owes its popularity to the fact that goats can thrive in diverse and changing environmental conditions, and that GM-based products are regarded as a gourmet food and health benefits of consuming GM have been defined [13]. Here we show that acceptance of GM is high and that GM is even slightly preferred over CM. Therefore, GM can be considered as both a nutritious and palatable choice for individuals at various age groups that incorporate milk in their diets.

## Figures and Tables

**Table 1:** CM and GM milk powder composition.

Sample	Protein	Fat	Carbohydrate (Lactose)	Ash	Moisture
CM	37.1	1.1	51	6.5	4.3
GM	36.1	0.9	49.9	9.5	3.6

**Table 2:** Composition of CM- and GM-enriched chow.

Base Skim Milk Powder Composition (1% Milk Fat)	Goat Skim Milk Powder (SMP)		Cow SMP	
	%	g/kg	%	g/kg
Protein	37.8	120	37.1	120
Fat	1.7	5.40	0.86	2.78
Ash	9.4	29.84	7.3	23.61
Moisture	4.1	13.02	4.2	13.58
Lactose	47	149.21	50.54	163.47
Total	100	317.46	100	323.45
	**Goat SMP**		**Cow SMP**	
**Skim Milk Chow** **—** **Ingredient**	g/kg		g/kg	
Goat SMP—to supply 12% protein	317.46			
Cow SMP—to supply 12% protein			323.45	
Vitamin mix	50.00		50.00	
Salt mix	50.00		50.00	
Corn oil (added to make 5% fat)	44.60		47.22	
Starch	447.15		452.80	
Lactose (added to make 16.5%)	15.79		1.53	
Cellulose	75.00		75.00	
Moisture				
	1000.00		1000.00	
**Target Dietary Content**				
Protein	12%		12%	
Fat	5%		5%	
Lactose	16.50%		16.50%	
Fiber	7.50%		7.50%	
Starch	44.7%		45.3%	
**Calories**				
Protein (4 cal/g)	48		48	
Fat (9 cal/g)	45		45	
Lactose (4 cal/g)	66		66	
Fiber (2 cal/g)	15		15	
Starch (4 cal/g)	179		181	
Calories/100 g	353		355	
**% calories**				
Protein	14%		14%	
Fat	13%		13%	
Lactose	19%		19%	
Fiber	4%		4%	
Starch	51%		51%	
**Skim Milk Powder (1% Milk Fat)**	**Goat SMP**		**Cow SMP**	
**Ingredients**	g/kg		g/kg	
Goat SMP	317.46			
Cow SMP			323.45	
Vitamin mix	50.00		50.00	
Salt mix	50.00		50.00	
Corn oil	44.60		47.22	
Starch	447.15		452.80	
Lactose	15.79		1.53	
Cellulose	75.00		75.00	
Moisture				
	1000.00		1000.00	
**Target Dietary Content**				
Protein	12%		12%	
Fat	5%		5%	
Lactose	16.50%		16.50%	
Fiber	7.50%		7.50%	
Starch	44.7%		45.3%	
**Calories**				
Protein (4 cal/g)	48		48	
Fat (9 cal/g)	45		45	
Lactose (4 cal/g)	66		66	
Fiber (2 cal/g)	15		15	
Starch (4 cal/g)	179		181	
Calories/100 g	353		355	
**% calories**				
Protein	14%		14%	
Fat	13%		13%	
Lactose	19%		19%	
Fiber	4%		4%	
Starch	51%		51%	
